# Studies of Microbiota Dynamics Reveals Association of “*Candidatus* Liberibacter Asiaticus” Infection with Citrus (*Citrus sinensis*) Decline in South of Iran

**DOI:** 10.3390/ijms19061817

**Published:** 2018-06-20

**Authors:** Alessandro Passera, Hamidreza Alizadeh, Mehdi Azadvar, Fabio Quaglino, Asma Alizadeh, Paola Casati, Piero A. Bianco

**Affiliations:** 1 Department of Agricultural and Environmental Sciences, University of Milan, 20133 Milan, Italy; alessandro.passera@unimi.it (A.P.); fabio.quaglino@unimi.it (F.Q.); paola.casati@unimi.it (P.C.); 2 Department of Plant Protection, Faculty of Agriculture, University of Jiroft, Jiroft 7867161167, Iran; hamid6948@gmail.com (H.A.); Alizadeh.b5273@gmail.com (A.A.); 3 Plant Protection Department, South Kerman Agricultural and Natural Resources Research and Education Center, Agricultural Research Education and Extension Organization, Jiroft 7867161167, Iran; mehdiazadvar@gmail.com

**Keywords:** citrus decline disease, *Citrus sinensis*, Bakraee, “*Candidatus* Liberibacter”, “*Candidatus* Phytoplasma”, microbiota

## Abstract

Citrus Decline Disease was recently reported to affect several citrus species in Iran when grafted on a local rootstock variety, Bakraee. Preliminary studies found “*Candidatus* Phytoplasma aurantifoliae” and “*Candidatus* Liberibacter asiaticus” as putative etiological agents, but were not ultimately able to determine which one, or if an association of both, were causing the disease. The current study has the aim of characterizing the microbiota of citrus plants that are either asymptomatic, showing early symptoms, or showing late symptoms through amplification of the V1–V3 region of 16S rRNA gene using an Illumina sequencer in order to (i) clarify the etiology of the disease, and (ii) describe the microbiota associated to different symptom stages. Our results suggest that liberibacter may be the main pathogen causing Citrus Decline Disease, but cannot rule out the possibility of phytoplasma being involved as well. The characterization of microbiota shows that the leaves show only two kinds of communities, either symptomatic or asymptomatic, while roots show clear distinction between early and late symptoms. These results could lead to the identification of bacteria that are related to successful plant defense response and, therefore, to immunity to the Citrus Decline Disease.

## 1. Introduction

Since 2010, a declining condition on trees belonging to different citrus species has widely appeared in various groves of the Southern Kerman region of Iran and has subsequently killed around 10% of cultivated citrus trees. This condition, called Citrus Decline Disease (CDD), causes symptoms that have been classified as (i) early CDD symptoms, which include leaves developing a pale green color, limited production of fresh sprouts, and in general a retardation of growth (Figure S1a), and (ii) late CDD symptoms which manifest as an evident tree decline along with reduction and decay of the root system (Figure S1b) [1]. Plants affected by CDD initially develop the milder, early symptoms and later develop the more severe late symptoms. Affected plants die approximately 5 years after the development of the first symptoms.

These symptoms, similar to those associated with obligate bacterial parasites, brought to the identification of putative etiological agents of the disease in both phytoplasma and liberibacter, as these microorganisms were detected by PCR in infected plants and not in healthy plants [1]. In particular, the putative pathogens were identified as “*Candidatus* Liberibacter asiaticus” and “*Candidatus* Phytoplasma aurantifolia”.

The liberibacter-associated citrus disease known as Huanglongbing (HLB) is also present in Iran. The disease was first detected from various locations in Sistan-Baluchistan and Hormozgan provinces of southern Iran on Valencia sweet orange trees (*Citrus Sinensis* (L.) Osbek) in 2009 [2].

Sour orange (*C. aurantium* L.), lime (*C. x aurantifolia* Swigle) and Bakraee (*C. reticulata* Blanco x *C. limettioides* Tan.) are the three most prevalent citrus rootstocks in Iran [3]. Bakraee in particular is being used extensively as a rootstock in the southern part of Iran for its shallow root system and tolerance to high pH [4]. Interestingly, new emerging citrus decline disease can be easily observed on several different citrus species aged six or more years old such as sweet orange, grapefruit (*C. x paradisi* Macfad), and mandarin (*C. reticulata* Blanco), but only when grafted on Bakraee. 

Diseases associated to phloem-limited pathogens, such as phytoplasma and liberibacter, are notoriously difficult to manage since there is no direct treatment that can be used against them [5], unlike other pathogens such as fungi that can be controlled through fungicides. For this reason, investigation of the precise etiology of these diseases, as well as of environmental conditions related to their epidemiology, can offer solutions for the management of the disease [6].

Several studies were carried out on HLB, a disease that shares the candidate etiological agent and host with CDD, and it is known that the disease causes a great restructuring of the microbiota of the host and it has been suggested that the dynamics of the bacterial microbiome can affect the concentration of “*Ca*. Liberibacter asiaticus”, which are correlated to symptom severity [7,8].

On the basis of these previous studies, it is plausible to hypothesize that a similar phenomenon might occur in the CDD-affected plants, and that the microbiota dynamics might therefore explain how the disease develops.

In the present study, the bacterial microbiota of asymptomatic plants, early symptomatic plants, and late symptomatic plants, determined both from leaf and root, was described and compared with the aims of (i) clearing the etiology of the disease and (ii) identifying the shifts in the microbial community linked to the development of the disease, in order to identify possible taxonomical units associated to a diseased or healthy state.

## 2. Results

### 2.1. Detection of Phytoplasma and Liberibacter in Plant Material

None of the samples from asymptomatic plants showed amplification when phytoplasma or liberibacter PCR assays were performed; on the contrary, samples from the early symptomatic or late symptomatic plants showed amplification for at least one of the putative pathogens. In particular, phytoplasma were detected in one out of two late symptomatic plants (LSP) leaves and one out of three early symptomatic plants (ESP) leaves; phytoplasma were also detected in two out of two LSP roots and one out of three ESP roots. Liberibacter were detected in all five symptomatic roots samples, regardless of symptom stage (Table 1). These amplified fragments were sequenced and the comparison with the database showed that the phytoplasma sequences belonged to the “*Ca*. P. aurantifolia” species while the liberibacter sequences belonged to the “*Ca*. L. asiaticus” species. 

### 2.2. Microbial Community in Iranian Citrus Plants

The MiSeq sequencing produced a total of 160,010 reads for asymptomatic plants (ASP) (84,588 leaf, 75,411 root), 283,715 reads of ESP (102,911 leaf, 180,804 root), and 144,744 reads for LSP (72,212 leaf, 72,532 root), allowing the description of 2762 operational taxonomic units (OTUs). After filtering, the remaining reads were 18,153 for ASP (7917 leaf, 10,236 root), 21,859 reads for ESP (7736 leaf, 13,853 root), and 76,385 reads for LSP (1588 leaf, 74,797 root), for a total of 2699 OTUs. For each plant sanitary status and organ, rarefaction curves were described (Figure S2).

**Figure 1:**
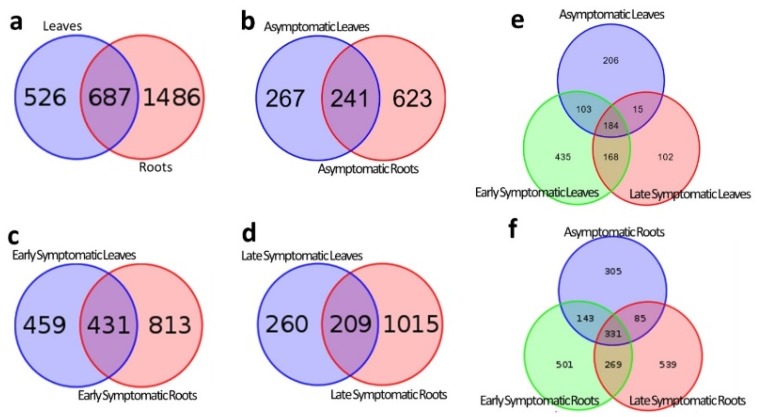
Graphs describing the different distribution of OTUs among different organs and/or sanitary statuses of examined plants: (**a**) Between all leaves and roots, regardless of sanitary status; (**b**) between leaves and roots of asymptomatic plants; (**c**) between leaves and roots of early symptomatic plants; (**d**) between leaves and roots of late symptomatic plants; (**e**) between the asymptomatic, early symptomatic, and late symptomatic plants at the leaves; (**f**) between the asymptomatic, early symptomatic, and late symptomatic plants at the roots.

Of these OTUs, most were exclusive to the root compartment (1486), followed by those shared among leaf and root (687), and the fewest were identified in leaf only (526) (Figure 1a). Still, these results indicate that relatively few bacterial OTUs are shared among the leaf and root compartments, identifying very diverse microbiota in the different organs. Considering the single sanitary statuses (ASP, ESP, or LSP), the trend is slightly different: While the number of root unique OTUs remains the highest, the leaf unique OTUs are higher than those shared between the two organs (Figure 1b–d). When considering the single plant organ (either leaves or roots), their differences appear clear. In the leaves, the highest amount of different OTUs is registered in ESP, followed by ASP, and lastly LSP; while there is a high amount of shared OTUs between ESP and ASP, ESP and LSP, and the three sanitary statuses, only few OTUs are common among ASP and LSP only (Figure 1e). In the roots the highest amount of different OTUs is found in LSP, followed by ESP, and by ASP; also in roots there is a high number of shared OTUs between the different sanitary statuses: ASP and LSP share the least amount while, the highest is the “core” among all three sanitary statuses (Figure 1f). Only 94 OTUs are shared among all sanitary statuses and plant compartments and could therefore be thought of as the ‘core’ microbiota of these plants. This core is constituted mostly by Firmicutes (45 OTUs), followed by Proteobacteria (19 OTUs), Bacterioidetes (11 OTUs), and Actinobacteria (9 OTUs) with the remaining 10 OTUs belonging to other phyla. It is interesting to notice that among this core microbiota are both OTUs belonging to “*Ca*. Liberibacter spp.” and “*Ca*. Phytoplasma spp”.

### 2.3. Bacterial Diversity among Different Organs and Sanitary Statuses

Bacterial diversity among the different organs and sanitary statuses was evaluated preliminary by comparing two indices, the Chao-1 and phylogenetic distance (PD) index (Table 2). From these indices it was possible to see that, in general, leaves have a less diverse and complex bacterial community compared to roots and that asymptomatic plants have a less complex bacterial community than symptomatic plants.

**Figure 2:**
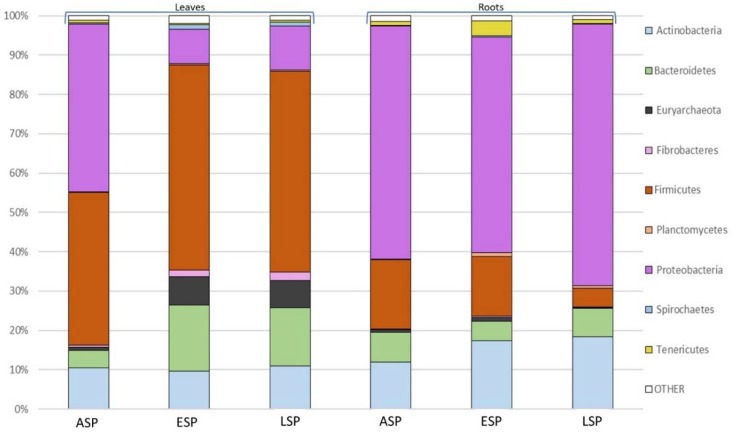
Graph describing the taxonomic distribution of OTUs among different organs (leaves or roots, indicated by brackets at the top) and/or sanitary statuses (asymptomatic (ASP), early symptomatic (ESP), or late symptomatic (LSP)) of examined plants at phylum level.

The first level of diversity analysis, performed at phylum level, was carried out to compare the samples grouped by sanitary status and organ (Figure 2).

**Figure 3:**
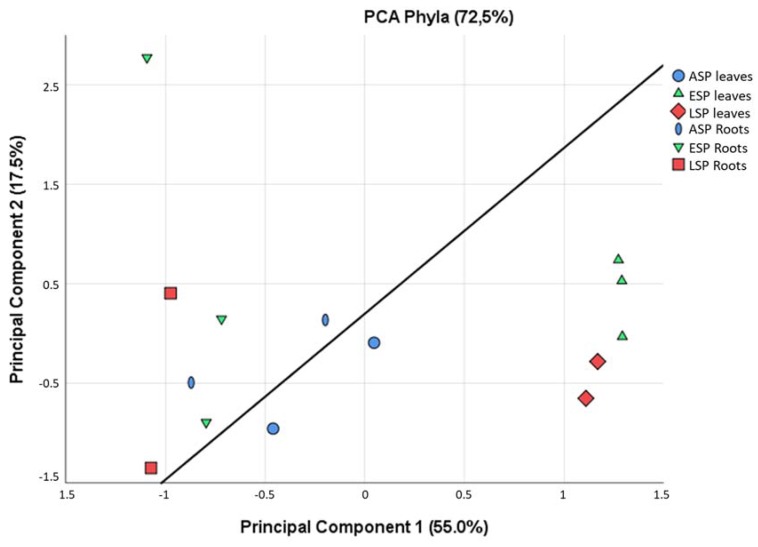
Principal Component Analysis calculated among all the samples, based on the relative abundance of OTUs in each phyla. The line was added to highlight the separation between leaves and roots.

While some similarities can be seen among all samples, such as the prevalence of three *phyla* among all others (Actinobacteria, Firmicutes, Proteobacteria), there are some differences that are highlighted already at this taxonomic level. For example, Fibrobacteres and Spirochaetes are relevant groups only in ESP and LSP leaves, and Planctomycetes are relevant only in ESP and LSP roots. In general, it can be said that the leaves are dominated by Firmicutes, while the roots are dominated by Proteobacteria, already depicting very different scenarios that are confirmed by a Principal Component Analysis (Figure 3). From this analysis it is evident that the leaves and roots contain a very different microbiota, which becomes even more different when the plants develop symptoms of CDD. For this reason, the subsequent microbiota analyses carried out in this study were done separately on the leaf and root compartment.

### 2.4. Metagenome-Based Diagnosis on Plant Material

**Figure 4:**
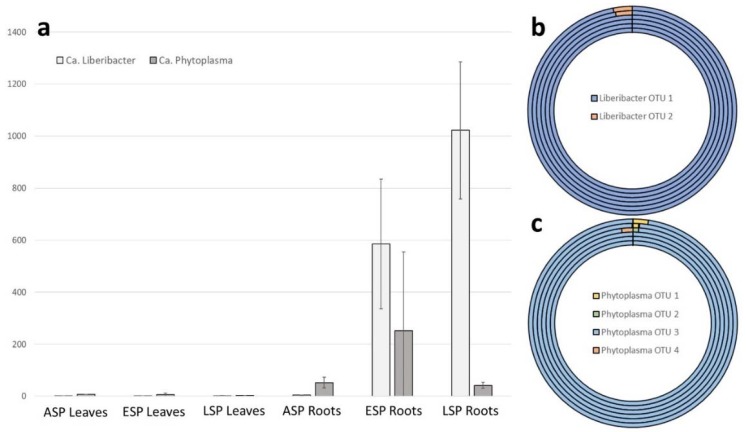
Graphs reporting the presence and abundance of pathogenic agents “*Ca*. Phytoplasma” and “*Ca*. Liberibacter” in the analyzed material according to metagenomic analyses: (**a**) Bar graph showing the average absolute abundance (number of OTUs) for each category of sample, with error bars indicating standard deviation among samples of the same category. Light grey bars indicate “*Ca*. Liberibacter” while dark grey bars indicate “*Ca*. Phytoplasma”; (**b**) circular graph indicating the relative abundance of different OTUs associated to “*Ca*. Liberibacter” in the different samples. Each circular bar represents a different category of sample as follow, from innermost circle to outermost: ASP leaf, ESP leaf, LSP leaf, ASP root, ESP root, LSP root; (**c**) circular graph indicating the relative abundance of different OTUs associated to “*Ca*. Phytoplasma” in the different samples. Each circular bar represents a different category of sample as follow, from innermost circle to outermost: ASP leaf, ESP leaf, LSP leaf, ASP root, ESP root, LSP root.

The reads obtained from the MiSeq analysis were searched specifically for OTUs assigned to the genera “*Ca*. Phytoplasma” and “*Ca*. Liberibacter”, in order to compare the results of this NGS technique to those obtained by regular PCR. OTUs belonging to both pathogens were found in all analyzed samples (Table 1), both symptomatic and asymptomatic, although the asymptomatic plants showed a lower amount of reads belonging to these bacteria (0 to 5 reads for liberibacter, 0 to 72 for phytoplasma), below the 1% threshold used to consider them relevant. On the other hand, both bacteria were present more consistently in symptomatic plants: in particular, while both ESP and LSP showed a high amount of reads belonging to liberibacter (0 to 1286 and 0 to 959 respectively), it is interesting to note that the LSP plants show a presence of phytoplasma comparable to that of ASP (0 to 54) while ESP have a much higher number of reads belonging to phytoplasma (0 to 707) (Figure 4a). It is also of note that both pathogens are found almost exclusively in the roots.

Other than the abundance of these pathogens, this analysis allowed to detect the presence of different OTUs belonging to liberibacter and phytoplasma, 2 and 4 respectively. Each of these genera has one dominant OTU that constitutes the entirety of the detected OTUs in asymptomatic plants and almost the entirety of detected OTUs in symptomatic plants (Figure 4b,c). It is interesting to note that the roots of symptomatic plants show the presence of an OTU for liberibacter and one for phytoplasma that are not present in healthy roots or in leaves. These OTUs represent a small percentage of those belonging to each pathogenic genus (approximately 2.75% for liberibacter and between 0.9 and 2.4 for phytoplasma).

### 2.5. Bacterial Diversity in Leaves: Shift of Community in Relation to Sanitary Status

The composition of the bacterial community associated with the leaves in the three sanitary statuses (ASP, ESP, and LSP) was compared at the family level (Table 3). Significant differences in relative abundance were identified in only 10 out of the 34 considered families. In particular, the Micrococcaeae, Gemellaceae, and Streptococcaeae were significantly more abundant in ASP than in ESP and LSP; while Coriobacteraceae, an unidentified family belonging to the Bacteriodales order, an unidentified family belonging to the Clostridiales order, Lachnospiraceae, Ruminococcaceae, Erysipelotrichaceae, and Desulfovibrionaceae families were significantly more abundant in ESP and LSP than in ASP.

**Figure 5:**
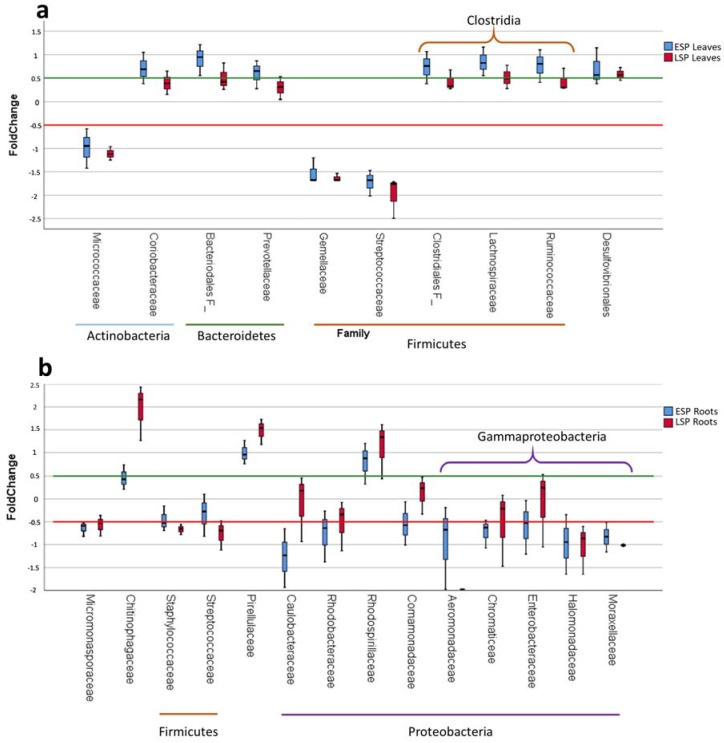
Box plots reporting the shift in absolute abundance of relevant taxonomical families in ESP and LSP samples compared to the ASP. Difference in abundance is expressed as fold change in a logarithmic scale (base 10). Median values above 0.5 or above −0.5 were considered relevant differences. (**a**) box plot showing the differences in leaves, highlighting with lines at the bottom families belonging to the same *phylum*, and with brackets on top those belonging to the same order; (**b**) box plot showing the differences in roots, highlighting with lines at the bottom families belonging to the same *phylum*, and with brackets on top those belonging to the same class.

The difference in the number of OTUs for these families, calculated as fold change, showed that the most significant shift in the communities from the asymptomatic to the symptomatic state can be identified in the change in composition in the Firmicutes phylum (Figure 5a): while the percentage of Firmicutes on the total bacteria is almost unchanged between ASP, ESP, and LSP, in ASP plants the Firmicutes are mostly Bacillales and Gemellales, while in ESP and LSP plants most of the Firmicutes belong to the Clostridiales order.

**Figure 6:**
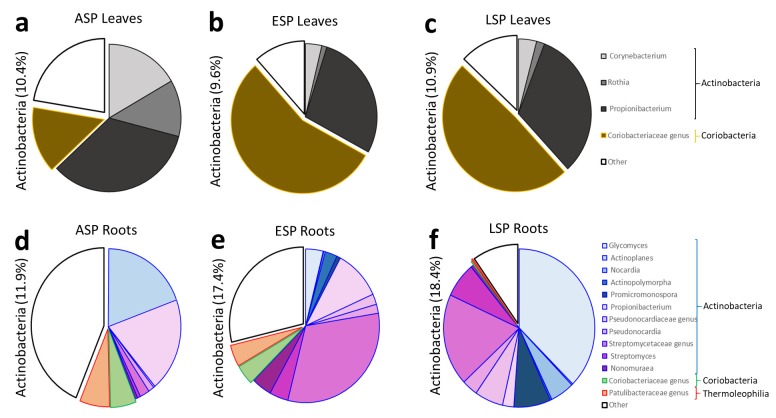
Pie graphs describing the relative abundance of genera in the Actinobacteria *phylum*. Between brackets is indicated the total share of the *phylum*, while each genus is represented by the size of the corresponding slice. (**a**) ASP leaves; (**b**) ESP leaves; (**c**) LSP leaves; (**d**) ASP roots; (**e**) ESP roots; (**f**) LSP roots.

**Figure 7:**
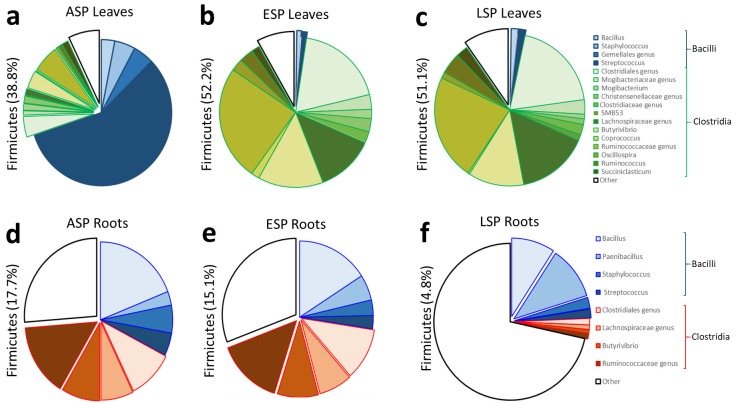
Pie graphs describing the relative abundance of genera in the Firmicutes *phylum*. Between brackets is indicated the total share of the *phylum*, while each genus is represented by the size of the corresponding slice. (**a**) ASP leaves; (**b**) ESP leaves; (**c**) LSP leaves; (**d**) ASP roots; (**e**) ESP roots; (**f**) LSP roots.

**Figure 8:**
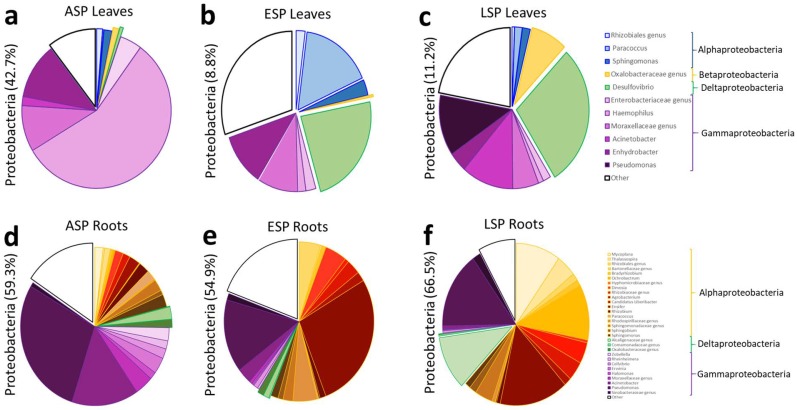
Pie graphs describing the relative abundance of genera in the Proteobacteria *phylum*. Between brackets is indicated the total share of the *phylum*, while each genus is represented by the size of the corresponding slice. (**a**) ASP leaves; (**b**) ESP leaves; (**c**) LSP leaves; (**d**) ASP roots; (**e**) ESP roots; (**f**) LSP roots.

These results are also reflected by the analysis at genus level carried out on the abundance of the genera detected in the three main *phyla* (Actinobactera, Firmicutes, Proteobacteria): the main shift detectable in the Actinobacteria regards the increase of Coriobacteriaceae at the expense of *Propriobacterium* spp. (Figure 6a–c); furthermore, the genus-level analysis confirms the shift that was detected at family-level from *Streptococcus* spp. to various genera of the Clostridiales family (Figure 7a–c); for the Proteobacteria, the shift in bacterial community saw the reduction of several genera in the Gammaproteobacteria and an increase in an unidentified genus in the Oxalobacteraceae family and in the *Desulfovibrio* genus (Figure 8a–c).

**Figure 9:**
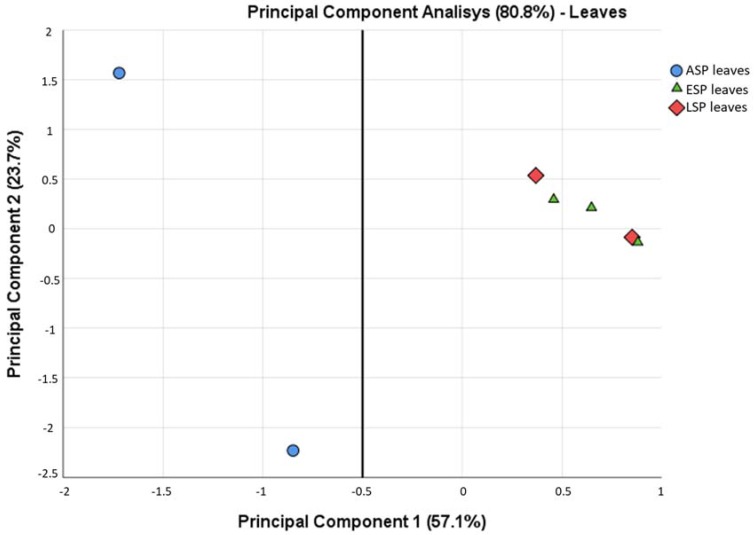
Principal Component Analysis calculated among the leaf samples, based on the relative abundance of OTUs in each family. The line was added to highlight the separation between the ASP leaf samples and the ESP/LSP leaf samples.

From all these analyses, no clear and significant difference could be identified between the bacterial communities in leaves of ESP and LSP, while the leaves of ASP differ from both. This consideration is supported also by the PCA calculated at a family level in leaves only (Figure 9) which clearly shows how the ASP samples are distinct from the ESP and LSP samples, which, instead, are not clearly separated. 

### 2.6. Bacterial Diversity in Roots: Shift of Community in Relation to Sanitary Status

The composition of the bacterial community associated to the roots in the three sanitary statuses (ASP, ESP, and LSP) was compared at the family level (Table 4). Significant differences in relative abundance were identified in 16 out of the 52 considered families. In particular, the Micrococcaeae, Micromonosporaceae, Staphyloccocaceae, Streptococcaeae, Caulobacteraceae, Rhodobacteraceae, Comamonadaceae, Aeromondaceae, Enterobacteriaceae, Halomonadaceae, and Moraxellaceae were significantly more abundant in ASP; Mycobateriaceae, Pirellulaceae, and Rhodospirillaceae were significantly more abundant in ESP; Chitinophagaceae was the only family over-represented in LSP samples.

The difference in the number of OTUs for these families, calculated as fold change, showed that the most significant shift in the communities from the asymptomatic to the symptomatic state can be identified in the change in the composition in the overall reduction in Firmicutes and Proteobacteria (Figure 5b): With the exception of Rhodospirillaceae, all relevant families of the Proteobacteria are much under-represented in ESP and LSP compared to ASP. In particular, Gammaproteobacteria are all greatly reduced, most of them reaching 10 times less abundance, and the Aeromonadaceae being almost 100 times less represented in LSP compared to ASP.

The analysis at genus level showed how the composition of the microbial community in the three main phyla (Actinobactera, Firmicutes, Proteobacteria) in ESP samples seemed to be intermediate between the ASP and LSP status: in the Actinobacteria community of ASP there is a large presence of lesser genera, and a relevant presence of Cortiobacteria and Thermoleophilia of unidentified genera (Figure 6d). In LSP there is way lesser abundance of these genera, while more than 30% of Actinobacteria belong to the Glycomyces genus (Figure 6f). The ESP plants still show a relevant abundance of Coriobacteria and Thermoleophilia, but also a higher abundance of Actinobacteria, with the appearance of the Glyomyces (Figure 6e). The investigation of the Firmicutes phylum at genus level reveals that the ASP and ESP samples show an almost identical composition of community (Figure 7d,e). The LSP plants instead have a much lower abundance of Firmicutes, furthermore most of them belong to less represented genera, with the the relative abundance of Bacilli remaining similar to ASP and LSP, but losing most of the genera belonging to Clostridia (Figure 7f). Conversely, the ESP and LSP samples are very similar regarding Proteobacteria, while, while the ASP is different. In the ASP plants most of the Proteobacteria belong to the Pseudomonas genus and, in general, Gammaproteobacteria constitute the majority of the community (Figure 8d), while in ESP and LSP the majority of the Proteobacteria belong to the Alphaproteobacteria, with the Rhizobia genus becoming the most represented one instead (Figure 8e,f).

**Figure 10:**
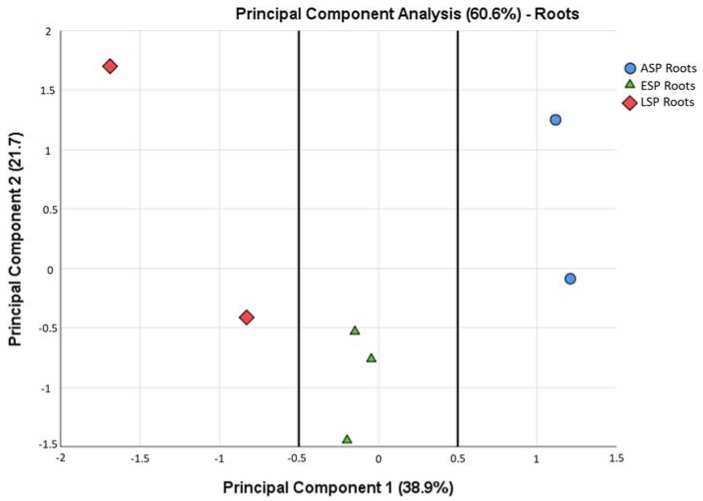
Principal Component Analysis calculated among the root samples, based on the relative abundance of OTUs in each family. The lines were added to highlight the separation between the ASP root samples, the ESP root samples, and the LSP root samples.

From all these analyses, it can be identified how the ESP have in fact an intermediate community between ASP and LSP, a consideration supported also by the PCA calculated at a family level in roots only (Figure 10), which clearly shows how the ASP, ESP, and LSP samples form their own distinct clusters. On the Component 1, the ESP cluster is found in the middle between the ASP and LSP clusters, while on the Component 2 the ESP cluster occupies the lower end of the scale, whereas the ASP and LSP clusters have similar placements, being closer to the positive values of the component.

## 3. Discussion

This study had the principal aim of investigating the bacterial community in Iranian citrus plants in order to describe the different sanitary statuses associated to Citrus Decline Disease and to clear the etiology of this disease.

All the positive detection results for phytoplasma and liberibacter obtained with PCR analyses were confirmed by metagenomics results; however, there are some samples, which are shown to contain pathogens by the metagenomics approach, that were negative to PCR assays, therefore indicating that the genomics technology is more sensitive than PCR [9].

Regarding the etiology of the disease, the results of the study offer some interesting perspectives, but no definitive answer: there is a higher abundance of liberibacter OTUs as the symptoms progress, while there is no particular correlation between symptoms progression and phytoplasma OTUs, as the highest amount is detected in ESP, which suggests that the main, or only, etiological agent of the disease is indeed “*Ca*. Liberibacter asiaticus”. Still, the role of phytoplasma cannot be ruled out entirely without further experimentations, since the disease could be caused by a synergistic effect of the two pathogens. In addition, the presence of specific OTUs, for both phytoplasma and liberibacter, in the ESP and LSP samples could suggest that it is not a species, but specific strains that, even at low concentrations, are the etiological agent or agents of the disease.

The presence of the pathogens in the roots, and not in the leaves, seems to suggest that the development of CDD is indeed related to the Bakraee rootstock. It is usually reported that ‘*Ca*. Liberibacter spp.’, despite being easily detected in roots even very early after infection [10], is present mostly in the leaves and not the roots of plants affected by the more common HLB disease [11]. Our results suggest that this is not the case for plants affected by CDD, in which the pathogen seems to replicate only in the rootstock and not in the scion.

The possible role of the rootstock in determining the development of the disease is further supported by some other information: (i) Bakraee variety is already reported as being extremely susceptible to infection by “*Ca*. P. aurantifolia” and its associated disease, Lime Witches’ Broom [12,13]; (ii) the higher abundance of liberibacter found in these roots is in accordance to a previous study [14] which found that this pathogen is more present in shallow, horizontally-developed roots than in vertical roots. As Bakraee is characterized by a shallow root system, this could contribute to the spread of liberibacter.

On the other hand, there is also to take into account that the leaves are more exposed to environmental factors, temperature in particular, than the roots. Since high temperatures can dramatically reduce the vitality of bacteria, it is possible that both phytoplasma and liberibacter can be found in leaves during colder months, following a seasonal fluctuation similarly as what already reported for “*Ca*. L. asiaticus” in association to HLB [15].

Since the pathogens, both liberibacter and phytoplasma, are localized in the roots of the symptomatic plants, their mode of transmission from one plant to another needs further investigation: it is possible that they are transmitted to the aerial part of the plant through the most well-known vectors for these kinds of pathogens, such as psyllids and leafhoppers, and then translocated inside the plant to reach the roots, as already reported for HLB [10], but with the pathogen remaining contained to the root compartment afterward; or that they are transmitted directly from root to root by some other, at the moment unknown, soil-borne vector. Another possibility is that the pathogen is transmitted by psyllids directly to the rootstock, as many of the infected plants show the presence of sprouts originating from Bakraee.

To clear the epidemiology of this disease, surveys should be carried out to detect the pathogens in putative insect vectors and reservoir plants in the area, as well as in other asymptomatic citrus plants grafted on different rootstocks from Bakraee.

Similarly as what reported for HLB, the presence of CDD symptoms causes a great restructuring of the microbiota, both in roots where the pathogens are found and in leaves where they are not present [11]. There are theories about how the restructuring of the microbiota happens in HLB, with the replication of liberibacter ousting other bacteria that would occupy the same ecological niche as the pathogen at a leaf level [16], and the lesser amount of nutrients translocated to the roots limiting the growth of endophytic bacteria while allowing the proliferation of opportunistic soil bacteria. The scenario is quite different in CDD. While an interaction with opportunistic soil bacteria can explain the differences at root level, where the pathogens proliferate, the shift at leaf level would be explained only by the change in nutrient content in the pale green canopy. It is unlikely that much of the restructuring detected at leaf level is caused by translocation of bacteria from the roots to the leaves, as the amount of shared OTUs between root and leaves in each sanitary status is quite low compared to that of unique OTUs in each compartment. It is possible that these additional OTUs are always present in leaves, regardless of sanitary status, but become much more abundant in the symptomatic plants, making them more easily detectable.

Since there are no curative treatments that can be used for either liberibacter- or phytoplasma-associated diseases, the perspective of harnessing the native endophytic microbiota of plants, that are key players in determining immunity or tolerance to diseases, to control the spread of diseases is particularly appealing [17]. In addition, the results obtained in this study confirm the general reports that asymptomatic trees are not entirely free from either liberibacter or phytoplasma [11], suggesting that the pathogens are kept below a threshold level needed for pathogenicity [7], or that more pathogenic strains are prevented from proliferating, by other components of the microbiota [18]. This last hypothesis is of particular interest in the light of the obtained results: both liberibacter and phytoplasma had specific OTUs that were found only in symptomatic plants, and in very low concentrations. This could suggest the hypothesis that there are hypervirulent strains that can give rise to the pathogenicity process, while the most common strains found in Iranian citrus, including asymptomatic plants, do not become pathogenic on their own.

The results of this study open possible perspectives for the research of biocontrol agents against this disease: focusing on the differences between the ASP and the symptomatic plants, it is noticeable that the symptomatic plants had lower abundance in bacteria of genera known for including biocontrol agents that are already in use, such as *Pseudomonas* and *Bacillus*. It is therefore possible that, by isolating endophytic bacteria from healthy plants, some cultivable bacteria presenting biocontrol traits that could be employed to protect citrus plants from the CDD disease could be identified. It is interesting to notice that, unlike what has been previously reported for HLB, there seems to be no major involvement of the *Burkholderia* genus, known to be a prevalent genus in biocontrol interaction [19] which is instead often reported as highly abundant in healthy citrus plants and less abundant in diseased plants [20].

Further studies should also be carried out to confirm these results on a higher number of samples, with sampling repeated on more time points, and investigating different citrus varieties, to better characterize the dynamics of the microbiota in relation to CDD in different seasons.

## 4. Materials and Methods

### 4.1. Sample Collection and Processing

The plant samples were collected from the leaves and roots of 8-year-old citrus plants from orchards located in Southern Kerman province during March 2017. Samples were collected from 2 asymptomatic plants (identified as ASP in the rest of the study), 3 plants showing early CDD symptoms (identified as ESP in the rest of the study) that showed the first symptoms in year 2015, and 2 plants showing late CDD symptoms (identified as LSP in the rest of the study) that showed the first symptoms in year 2013. For all these plants, leaf material came from the scion (*Citrus sinensis* L. Osbeck), sampled at one meter from the ground, while root material came from the local rootstock variety, Bakraee. Briefly, the tissues samples were transferred into the sterile biosafety bags, stored in ice boxes and transferred to the laboratory. After washing each sample under running tap water to remove soil particles (5 min), the samples were cut into 3–4 pieces (3–4 cm each) washed with sterile distilled water, and allowed to drain. Tissues were immersed separately in 70% ethanol (3 min), followed by sodium hypochlorite (2%) solution (1 and 3 min for leaves and roots, respectively), and into 70% ethanol (30 s). The samples were rinsed five times in sterile distilled water and were allowed to drain. To verify the accuracy of the surface sterilization procedure, the last rinsing water was inoculated onto nutrient agar sucrose plates and after any bacteria growth in the control agar plates, the sample discarded [21].

DNA was extracted from these samples, following the protocol described in [22].

### 4.2. Detection of Phytoplasma and Liberibacter in Plant Material

To preliminarily assess and identify the possible presence of phytoplasma and liberibacter species, nested PCR amplification was carried out using the universal primers and set of conditions that amplify portions of the 16S rRNA bacterial gene, as described in [1]. The presence of phytoplasmas was assessed using the P1/P7 primer pair in direct PCR, followed by the R16F2n/R16R2 primer pair in nested reaction (Table S1, [23]), while the presence of ‘*Ca*. L. asiaticus’ was assessed using the FD1/RP1 and OI1/OI2c universal primer pairs (Table S1, [24,25]). The amplified fragments were sent to an external service (Eurofins, Hamburg, Germany) for sequencing. Nucleotide sequences were assembled by the Contig Assembling Program in the software BioEdit version 7.2.6 [26]. The obtained nucleotide sequences were compared with related sequences based on the nBlast analysis software available at NCBI GenBank to search for the most similar sequences.

### 4.3. Microbiota Sequencing and OTU Determination

To DNA from sampled citrus plants was sent to an external service (Personal Genomics, Verona (VR), Italy) for sequencing of the hypervariable V1–V3 region of the 16S rRNA (Table S1, [27]) gene using a MiSeq1000 sequencer. The obtained reads (deposited in EMBL-ENA under accession number PRJEB26999; Available online: http://www.ebi.ac.uk/ena/data/view/PRJEB26999) were analyzed using the QIIME pipeline [28] in order to assign them to OTUs and determine the richness of species in the different samples. Reads that mapped on plant-derived sequences (mitochondria, chloroplasts), and reads with low quality, were filtered out. Alpha diversity was calculated for each sample using the Chao-1 and PD indices.

### 4.4. Microbiota Analysis

The sequencing data were analyzed in different ways. A first stage of analysis included the description of rarefaction curves to determine the reliability of the sequencing and the identification of OTUs that were unique to certain sanitary statuses and/or organs, opposed to shared or “core” OTUs. Then, OTUs belonging to the putative pathogens were searched/selected and quantified among samples of different sanitary statuses and/or organs.

The composition of the bacterial community, expressed as relative abundance (1% cutoff threshold), was defined at the *phylum* and family level for all sanitary statuses and organs analyzed. Furthermore, composition of the community was also analyzed at a genus level, only for the bacteria belonging to the three *phyla* that were consistently more represented among the samples (Actinobacteria, Firmicutes, Proteobacteria). 

The difference of relative abundance for relevant taxonomical units (either at *phylum*, family, or *genus* level) in different samples (ASP, ESP, LSP) was evaluated through one-way ANOVA performed on the percentage of abundance of these taxonomical units using the SPSS statistical package for Windows v. 23.0 (SPSS Inc., Chicago, IL, USA).

The number of OTUs was compared as fold change (Log10 base) between families that showed significant differences in relative abundance. For this analysis, changes were considered relevant when the fold change was above 0.5 or below −0.5. The relative abundance of OTUs at phylum and family level was also used to calculate the PCA among leaf samples and root samples.

## Figures and Tables

**Table 1:** Table reporting the results of detection of phytoplasma and liberibacter in the analyzed samples, both with PCR and with next generation sequencing (NGS).

Sample		PCR		NGS ^1^	
		Phytoplasma	Liberibacter	Phytoplasma	Liberibacter
1 (ASP)	Leaf	−	−	+	+
	Root	−	−	+	+
2 (ASP)	Leaf	−	−	+	+
	Root	−	−	+	+
3 (ESP)	Leaf	−	−	−	−
	Root	+	+	+++	+++
4 (ESP)	Leaf	+	−	+	−
	Root	−	+	+	++
5 (ESP)	Leaf	−	−	+	+
	Root	−	+	+	++
6 (LSP)	Leaf	−	−	+	+
	Root	+	+	+	+++
7 (LSP)	Leaf	+	−	+	−
	Root	+	+	+	+++

^1^ NGS data: “−” = 0 operational taxonomic units (OTUs), “+” = 1–100 OTUs, “++” = 101–500 OTUs, “+++” = 501+ OTUs.

**Table 2:** Table reporting the values of Chao-1 and PD indexes, and their standard deviation, for the different categories of simples.

Sample Category		Chao-1	PD
ASP	Leaf	496 ± 72	31.6 ± 3.8
	Root	765 ± 40	42.5 ± 4.3
ESP	Leaf	741 ± 113	42.5 ± 6.3
	Root	1058 ± 276	53.2 ± 11.3
LSP	Leaf	572 ± 60	33.3 ± 3.1
	Root	1187 ± 351	52.6 ± 11.4

**Table 3:** Taxonomic relations and relative abundance, with standard deviation, of OTUs at family level in leaves. *p* value is indicated for One-Way ANOVA, followed by Tukey’s exact post-hoc test, carried out to determine significant differences between abundances. Significant differences are highlighted by bold text, and * indicates which result is different from the others.

Phylum	Class	Order	Family	ASP	ESP	LSP	*p*
Actinobacteria	Actinobacteria	Actinomycetales	Corynebacteriaceae	1.74 ± 0.88	0.43 ± 0.06	0.35 ± 0.20	0.34
			**Micrococcaceae**	1.84 ± 0.31 *	**0.28 ± 0.12**	**0.25 ± 0.19**	**0.02**
			Propionibacteriaceae	3.55 ± 1.41	3.04 ± 0.87	2.69 ± 1.11	0.85
	Coriobacteriia	Coriobacteriales	**Coriobacteriaceae**	1.54 ± 0.71 *	**5.91 ± 0.78**	**5.85 ± 0.59**	**0.05**
Bacteroidetes	Bacteroidia	Bacteriodales		1.77 ± 0.76 *	**10.29 ± 1.10**	**11.39 ± 0.48**	**0.00**
			[Paraprevotellaceae]	0.48 ± 0.22	0.85 ± 0.15	0.87 ± 0.24	0.47
			**Prevotellaceae**	0.55 ± 0.06 *	**3.09 ± 0.35**	**2.99 ± 0.05**	**0.02**
	Flavobacteria	Flavobacteriales	[Weeksellaceae]	1.12 ± 0.68	0.14 ± 0.10	0.15 ± 0.12	0.37
Euryarchaeota	Methanobacteria	Methanobacteriales	Methanobacteriaceae	0.64 ± 0.08	4.52 ± 0.86	4.76 ± 0.84	0.05
	Thermoplasmata	E2	[Methanomassiliicoccaeae]	0.15 ± 0.00	2.56 ± 0.58	2.52 ± 0.46	0.07
Fibrobacteres	Fibrobacteria	Fibrobacterales	Fibrobacteraceae	0.54 ± 0.26	1.87 ± 0.43	1.64 ± 0.53	0.12
Firmicutes	Bacilli	Bacillales	Bacillaceae	1.27 ± 0.68	0.16 ± 0.12	0.14 ± 0.10	0.31
			Staphylococcaceae	1.85 ± 1.01	0.61 ± 0.31	0.51 ± 0.23	0.49
		Gemellales	**Gemellaceae**	1.89 ± 0.27 *	**0.04 ± 0.05**	**0.07 ± 0.09**	**0.01**
		Lactobacillales	**Streptococcaceae**	22.45 ± 6.07 *	**0.63 ± 0.33**	**0.53 ± 0.19**	**0.04**
	Clostridia	Clostridiales		1.77 ± 0.46 *	**9.91 ± 0.51**	**9.85 ± 0.78**	**0.00**
			[Mogibacteriaceae]	0.92 ± 0.51	2.45 ± 0.26	2.70 ± 0.19	0.07
			Christensenellaceae	0.60 ± 0.32	1.13 ± 0.35	1.47 ± 0.41	0.20
			Clostridiaceae	0.03 ± 0.02	1.87 ± 0.62	1.59 ± 0.50	0.12
			**Lachnospiraceae**	2.33 ± 0.69 *	**15.87 ± 1.99**	**16.03 ± 1.69**	**0.01**
			**Ruminococcaceae**	2.94 ± 1.01 *	**15.36 ± 0.81**	**15.92 ± 1.22**	**0.00**
			Veillonellaceae	1.01 ± 0.25	1.39 ± 0.40	1.12 ± 0.36	0.45
	Erysipelotrichi	Erysipelotrichales	**Erysipelotrichaceae**	0.41 ± 0.24 *	**1.48 ± 0.19**	**1.51 ± 0.36**	**0.00**
Proteobacteria	Alphaproteobacteria	Rhodobacterales	Rhodobacteraceae	0.38 ± 0.16	1.24 ± 1.25	1.81 ± 2.11	0.66
		Sphingomonadales	Sphingomonadaceae	1.29 ± 0.78	0.78 ± 0.25	0.95 ± 0.23	0.71
	Betaproteobacteria	Burkholderiales	Comamonadaceae	0.80 ± 0.39	0.24 ± 0.06	0.23 ± 0.11	0.37
			Oxalobacteraceae	0.55 ± 0.35	0.59 ± 0.67	0.07 ± 0.09	0.41
	Deltaproteobacteria	Desulfovibrionales	**Desulfovibrionaceae**	0.35 ± 0.08 *	**2.74 ± 0.70**	**2.13 ± 0.59**	**0.02**
	Gammaproteobacteria	Enterobacteriales	Enterobacteriaceae	2.53 ± 1.28	0.35 ± 0.30	0.33 ± 0.32	0.29
		Pasteurellales	Pasteurellaceae	24.38 ± 15.64	0.20 ± 0.12	0.24 ± 0.16	0.32
		Pseudomonadales	Moraxellaceae	4.93 ± 2.64	1.49 ± 1.11	0.83 ± 0.24	0.46
			Pseudomonadaceae	4.99 ± 2.71	1.13 ± 0.59	0.95 ± 0.93	0.39
Spirochaetes	Spirochaetes	Spirochaetales	Spirochaetaceae	0.34 ± 0.18	1.01 ± 0.37	0.99 ± 0.20	0.50
Tenericutes	Mollicutes	Acholeplasmatales	Acholeplasmataceae	0.67 ± 0.37	0.35 ± 0.12	0.33 ± 0.22	0.72
Other				7.39 ± 3.15	6.05 ± 0.30	6.24 ± 0.33	

**Table 4:** Taxonomic relations and relative abundance, with standard deviation, of OTUs at family level in roots. *p* value is indicated for One-Way ANOVA, followed by Tukey’s exact post-hoc test, carried out to determine significant differences between abundances. Significant differences are highlighted by bold text, and superscript letters (^a,b^) indicate which results are different, while both letters (^a,b^) indicate intermediate results.

Phylum	Class	Order	Family	ASP	ESP	LSP	*p*
Actinobacteria	Actinobacteria	Actinomycetales	Glycomycetaceae	0.01 ± 0.01	0.67 ± 0.88	5.64 ± 5.35	0.27
			**Micrococcaceae**	**0.85 ± 0.19 ^a^**	**0.21 ± 0.14 ^b^**	**0.17 ± 0.10 ^b^**	**0.02**
			**Micromonosporaceae**	**2.46 ± 0.28 ^a^**	**0.73 ± 0.23 ^b^**	**0.47 ± 0.30 ^b^**	**0.00**
			**Mycobacteriaceae**	**0.33 ± 0.11 ^b^**	**2.04 ± 0.68 ^a^**	**0.56 ± 0.49 ^ab^**	**0.05**
			Nocardiaceae	0.01 ± 0.01	0.09 ± 0.04	0.83 ± 0.81	0.27
			Nocardioidaceae	0.68 ± 0.04	0.87 ± 1.00	0.46 ± 0.26	0.81
			Promicromonosporaceae	0.00 ± 0.00	0.19 ± 0.25	1.20 ± 1.13	0.29
			Propionibacteriaceae	2.35 ± 0.29	1.81 ± 0.65	0.72 ± 0.40	0.06
			Pseudonocardiaceae	0.24 ± 0.01	0.72 ± 0.64	1.76 ± 1.52	0.39
			Streptomycetaceae	0.34 ± 0.00	6.17 ± 2.77	5.06 ± 0.54	0.05
			Streptosporangiaceae	0.06 ± 0.04	0.87 ± 1.01	0.27 ± 0.20	0.47
	Coriobacteriia	Coriobacteriales	Coriobacteriaceae	0.84 ± 0.52	0.85 ± 0.28	0.22 ± 0.21	0.31
	Thermoleophilia	Solirubrobacterales	Patulibacteraceae	0.80 ± 0.08	0.79 ± 0.53	0.20 ± 0.20	0.23
Bacteroidetes	[Saprospirae]	[Saprospirales]	**Chitinophagaceae**	**0.21 ± 0.06 ^b^**	**0.79 ± 0.26 ^b^**	**2.96 ± 0.80 ^a^**	**0.01**
	Bacteroidia	Bacteriodales		1.78 ± 1.18	1.86 ± 0.43	0.41 ± 0.48	0.28
			Prevotellaceae	0.57 ± 0.34	0.56 ± 0.21	0.12 ± 0.15	0.28
	Flavobacteria	Flavobacteriales	[Weeksellaceae]	1.49 ± 0.23	0.17 ± 0.09	1.90 ± 1.77	0.34
			Flavobacteriaceae	1.77 ± 0.29	0.78 ± 0.55	1.12 ± 0.38	0.19
Euryarchaeota	Methanobacteria	Methanobacteriales	Methanobacteriaceae	0.67 ± 0.45	0.49 ± 0.08	0.13 ± 0.12	0.32
Firmicutes	Bacilli	Bacillales	Bacillaceae	3.71 ± 0.44	3.40 ± 1.74	1.26 ± 0.72	0.17
			Paenibacillaceae	0.57 ± 0.01	1.00 ± 0.64	0.71 ± 0.37	0.66
			**Staphylococcaceae**	**1.06 ± 0.15 ^a^**	**0.50 ± 0.18 ^b^**	**0.19 ± 0.12 ^b^**	**0.01**
		Lactobacillales	**Streptococcaceae**	**0.84 ± 0.19 ^a^**	**0.47 ± 0.18 ^ab^**	**0.19 ± 0.12 ^b^**	**0.03**
	Clostridia	Clostridiales		1.81 ± 1.14	1.68 ± 0.43	0.39 ± 0.43	0.28
			Lachnospiraceae	3.22 ± 2.11	2.73 ± 0.65	0.63 ± 0.70	0.30
			Ruminococcaceae	3.14 ± 2.05	2.77 ± 0.70	0.61 ± 0.72	0.30
Planctomycetes	Planctomycetia	Pirellulales	**Pirellulaceae**	**0.05 ± 0.04 ^b^**	**0.83 ± 0.20 ^a^**	**0.56 ± 0.20 ^ab^**	**0.01**
Proteobacteria	Alphaproteobacteria	Caulobacterales	**Caulobacteraceae**	**1.75 ± 0.50 ^a^**	**0.11 ± 0.10 ^b^**	**0.22 ± 0.09 ^b^**	**0.01**
		Kiloniellales	Kiloniellaceae	0.17 ± 0.10	0.00 ± 0.00	5.25 ± 5.15	0.25
		Rhizobiales		0.74 ± 0.01	2.55 ± 0.83	2.85 ± 1.40	0.19
			Bartonellaceae	0.00 ± 0.00	0.16 ± 0.20	0.82 ± 0.75	0.28
			Bradyrhizobiaceae	0.38 ± 0.09	0.55 ± 0.11	0.92 ± 0.54	0.37
			Brucellaceae	0.31 ± 0.11	0.53 ± 0.21	6.03 ± 5.74	0.27
			Hyphomicrobiaceae	1.52 ± 0.09	4.56 ± 1.48	4.04 ± 1.59	0.15
			Phyllobacteriaceae	0.08 ± 0.02	0.41 ± 0.11	0.61 ± 0.36	0.18
			Rhizobiaceae	4.19 ± 0.02	19.23 ± 5.85	15.49 ± 6.41	0.08
		Rhodobacterales	**Rhodobacteraceae**	**1.90 ± 0.04 ^a^**	**0.44 ± 0.21 ^b^**	**0.17 ± 0.09 ^b^**	**0.00**
		Rhodospirillales	**Rhodospirillaceae**	**0.38 ± 0.08 ^b^**	**3.06 ± 0.89 ^a^**	**1.35 ± 0.57 ^ab^**	**0.02**
		Sphingomonadales	Sphingomonadaceae	3.62 ± 0.18	2.74 ± 0.44	4.40 ± 0.80	0.08
	Betaproteobacteria	Burkholderiales	Alcaligenaceae	0.09 ± 0.04	0.13 ± 0.09	5.86 ± 5.79	0.26
			**Comamonadaceae**	**2.61 ± 0.27 ^a^**	**1.01 ± 0.45 ^b^**	**0.79 ± 0.14 ^b^**	**0.00**
			Oxalobacteraceae	1.49 ± 0.25	1.40 ± 0.77	0.44 ± 0.32	0.20
	Gammaproteobacteria	Aeromonadales	**Aeromonadaceae**	**1.87 ± 0.11 ^a^**	**0.68 ± 0.45 ^b^**	**0.14 ± 0.18 ^b^**	**0.00**
		Alteromonadales	**[Chromatiaceae]**	**1.17 ± 0.22 ^a^**	**0.25 ± 0.02 ^b^**	**0.47 ± 0.38 ^b^**	**0.05**
			Alteromonadaceae	1.19 ± 0.26	0.53 ± 0.58	0.59 ± 0.06	0.27
		Enterobacteriales	**Enterobacteriaceae**	**2.89 ± 0.27 ^a^**	**1.08 ± 0.61 ^b^**	**0.58 ± 0.27 ^b^**	**0.00**
		Oceanospilillales	**Halomonadaceae**	**0.89 ± 0.26 ^a^**	**0.19 ± 0.13 ^b^**	**0.10 ± 0.07 ^b^**	**0.02**
		Pseudomonadales	**Moraxellaceae**	**11.34 ± 2.37 ^a^**	**2.21 ± 0.64 ^b^**	**0.81 ± 0.49 ^b^**	**0.00**
			Pseudomonadaceae	16.96 ± 2.99	8.35 ± 3.02	9.95 ± 1.61	0.06
		Xanthomonadales	Sinobacteraceae	0.58 ± 0.20	0.87 ± 0.26	1.27 ± 0.84	0.54
			Xanthomonadaceae	0.55 ± 0.11	0.76 ± 0.29	0.31 ± 0.15	0.20
Tenericutes	Mollicutes	Acholeplasmatales	Acholeplasmataceae	1.00 ± 0.07	3.90 ± 4.45	0.99 ± 0.97	0.52
Other				12.45 ± 1.60	10.24 ± 0.28	6.81 ± 1.63	

## Supplementary Materials

Supplementary materials can be found at http://www.mdpi.com/1422-0067/19/6/1817/s1.

## Abbreviations

CDD	Citrus Decline Disease
HLB	Huanglongbing
ASP	Asymptomatic plants
ESP	Early symptomatic plant
LSP	Late symptomatic plant
