# Supplementary material for: Studies of Microbiota Dynamics Reveals Association of “Candidatus Liberibacter Asiaticus” Infection with Citrus (Citrus sinensis) Decline in South of Iran
Source: Int J Mol Sci. 2018 Jun 20;19(6):1817. doi: 10.3390/ijms19061817 (PMC6032414; doi:10.3390/ijms19061817)
Supplement: Supplementary file 1 [file ijms-19-01817-s001.zip › Table S1.pdf]

Table S1. Table reporting the primers used in the study, their sequence and source reference.

Primer	Sequence	Reference
P1	5′-AAGAGTTTGATCCTGGCTCAGGATT-3′	Lee *et al.*, 1998 [23]
P7	5′-CGTCCTTCATCGGCTCTT-3′	Lee *et al.*, 1998 [23]
R16F2n	5′-GAAACGACTGCTAAGACTGG-3′	Lee *et al.*, 1998 [23]
R16R2	5′-TGACGGGCGGTGTGTACAAACCCCC-3′	Lee *et al.*, 1998 [23]
FD1	5′-AGAGTTTGATCCTGGCTCAG-3′	Akhtar *et al.*, 2008 [24]
RP1	5′-ACGG(ACT)TACCTTGTTACGACTT-3′	Akhtar *et al.*, 2008 [24]
OI1	5′-TCGGCCGCCCTTCGAAACCCAT-3′	Jagoueix *et al.*, 1994 [25]
OI2c	5′-TCGGCCGCCCTTCGAAACCCAT-3′	Jagoueix *et al.*, 1994 [25]
V1	5′-CCTACGGGAGGCAGCAG-3′	Muyzer *et al.*, 1993 [26]
V3	5′-ATTACCGCGGCTGCTG-3′	Muyzer *et al.*, 1993 [26]
