# Supplementary figures and images for: Studies of Microbiota Dynamics Reveals Association of “Candidatus Liberibacter Asiaticus” Infection with Citrus (Citrus sinensis) Decline in South of Iran
Source: Int J Mol Sci. 2018 Jun 20;19(6):1817. doi: 10.3390/ijms19061817 (PMC6032414; doi:10.3390/ijms19061817)

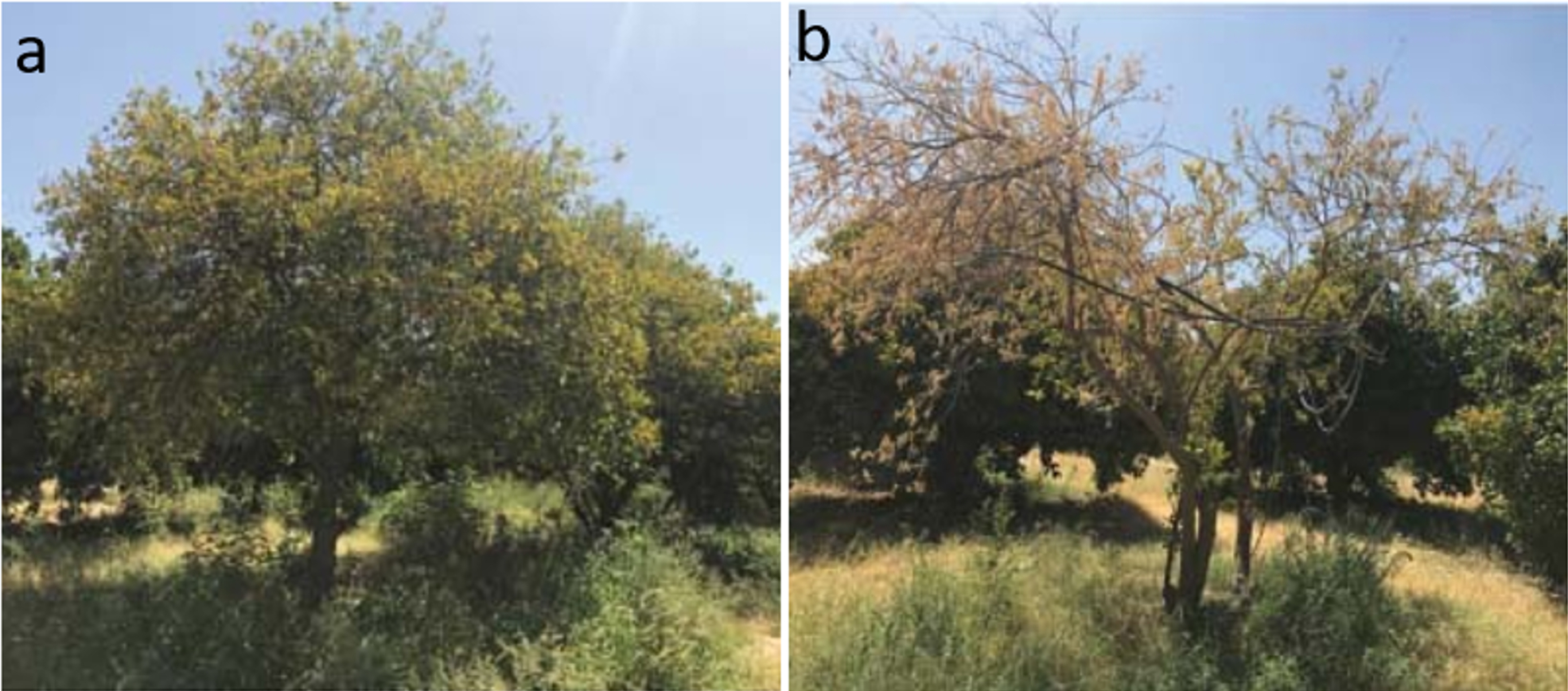

Supplement: Supplementary file 1 [file ijms-19-01817-s001.zip › Figure S1.jpg]

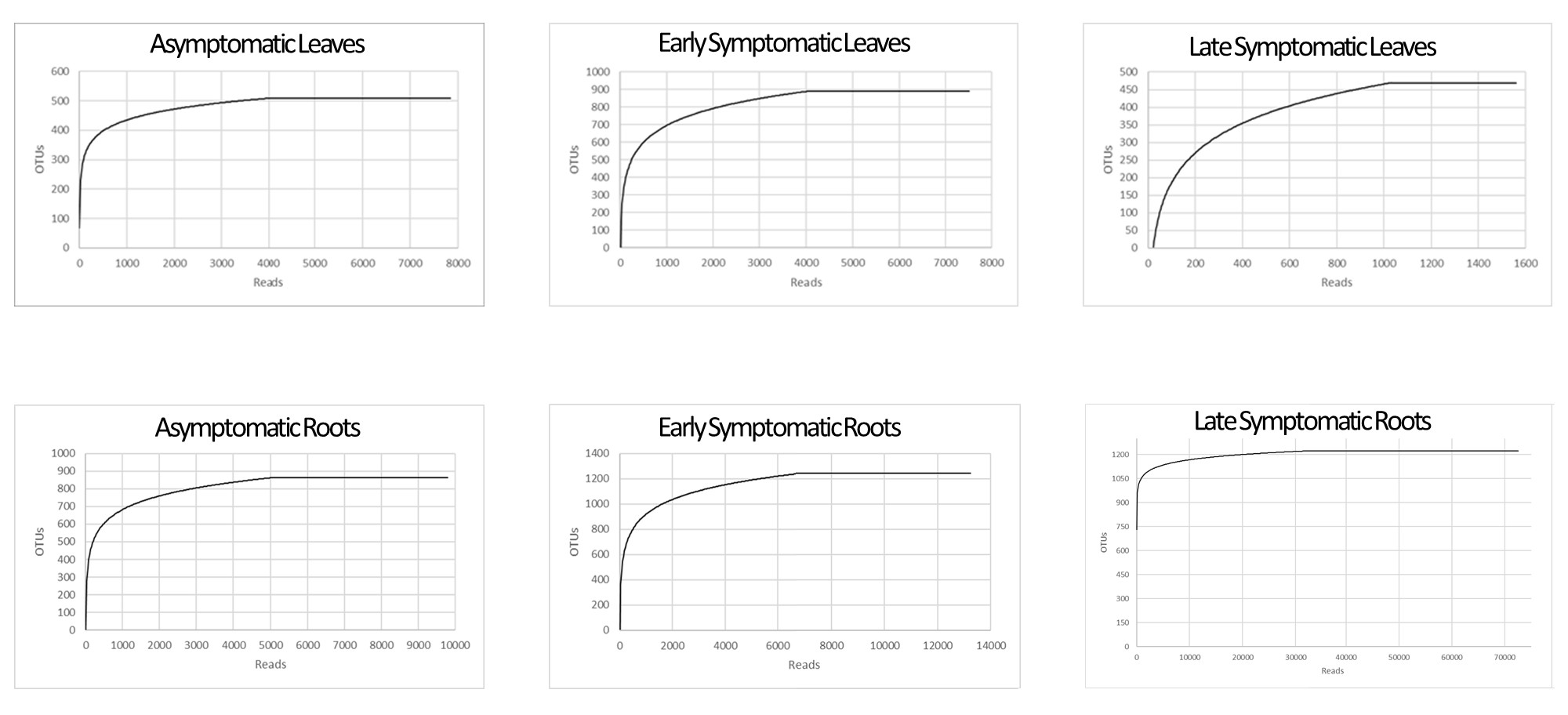

Supplement: Supplementary file 1 [file ijms-19-01817-s001.zip › Figure S2.jpg]
